# Equations for Solar Tracking

**DOI:** 10.3390/s120404074

**Published:** 2012-03-27

**Authors:** Alexis Merlaud, Martine De Mazière, Christian Hermans, Alain Cornet

**Affiliations:** 1 Belgian Institute for Space Aeronomy, Avenue Circulaire 3, 1180 Brussels, Belgium; E-Mails: martine@aeronomie.be (M.D.M.); christh@oma.be (C.H.); 2 Institute of Condensed Matter and Nanosciences, UCL, Chemin du Cyclotron 2, 1348 Louvain-La-Neuve, Belgium; E-Mail: alain.cornet@uclouvain.be

**Keywords:** solar tracker, Fourier transform infrared spectrometry, algorithms

## Abstract

Direct sunlight absorption by trace gases can be used to quantify them and investigate atmospheric chemistry. In such experiments, the main optical apparatus is often a grating or a Fourier transform spectrometer. A solar tracker based on motorized rotating mirrors is commonly used to direct the light along the spectrometer axis, correcting for the apparent rotation of the Sun. Calculating the Sun azimuth and altitude for a given time and location can be achieved with high accuracy but different sources of angular offsets appear in practice when positioning the mirrors. A feedback on the motors, using a light position sensor close to the spectrometer, is almost always needed. This paper aims to gather the main geometrical formulas necessary for the use of a widely used kind of solar tracker, based on two 45° mirrors in altazimuthal set-up with a light sensor on the spectrometer, and to illustrate them with a tracker developed by our group for atmospheric research.

## 1. Introduction

Spectroscopic analyses of direct incident sunlight are commonly used in atmospheric research. Such experiments make use of the Sun as a light source to quantify molecular absorptions in the atmosphere and then retrieve trace gas abundances. Stratospheric ozone [1] and greenhouse gases [2] are routinely measured with this technique from ground-based Fourier transform infrared (FTIR) spectrometers, e.g., within the Network for the Detection of Atmospheric Composition Change (NDACC, http://www.ndacc.org/). In the UV-visible range, light scattering is more important and enables spectroscopic studies of the atmosphere in other geometries such as zenith measurements [3]. However, direct sunlight is also used [4,5], its unique and unambiguous light path making it advantageous for some applications [6]. Beside the spectrometer, the main part of the involved apparatus in direct sunlight spectrometry is the solar tracker, required to compensate for the Sun's diurnal motion.

Several kinds of trackers, sometimes referred to as heliostats, are used for atmospheric spectrometry, based on setups of one or several rotating mirrors. Some of them are equatorially mounted, like in Table Mountain Facility [4] or Harestua [7]. In this case, one rotational axis is parallel to the Earth's axis. It enables a high tracking accuracy without a computer, since only one axis has to be driven at the Earth's rotation speed. To our knowledge, it is the only setup working without feedback on the Sun's position. On the other hand, equatorial mounts are large, need to be aligned accurately and their mechanical design is difficult. Most of the trackers used today are controlled by a computer enabling remote operation and automation. The computer first calculates the Sun position, moves the mirrors to point to the Sun and then controls these mirrors to optimize the signal on some kind of light sensor. For some trackers, the light sensor is attached to the moving part, whether it is a single mirror [8] or a mount of two mirrors [9]. Compared to the solution presented below, the retroaction is simplified. The drawback is that the tracking is done some meters away from the spectrometer and is thus less accurate and stable.

**Figure 1.**
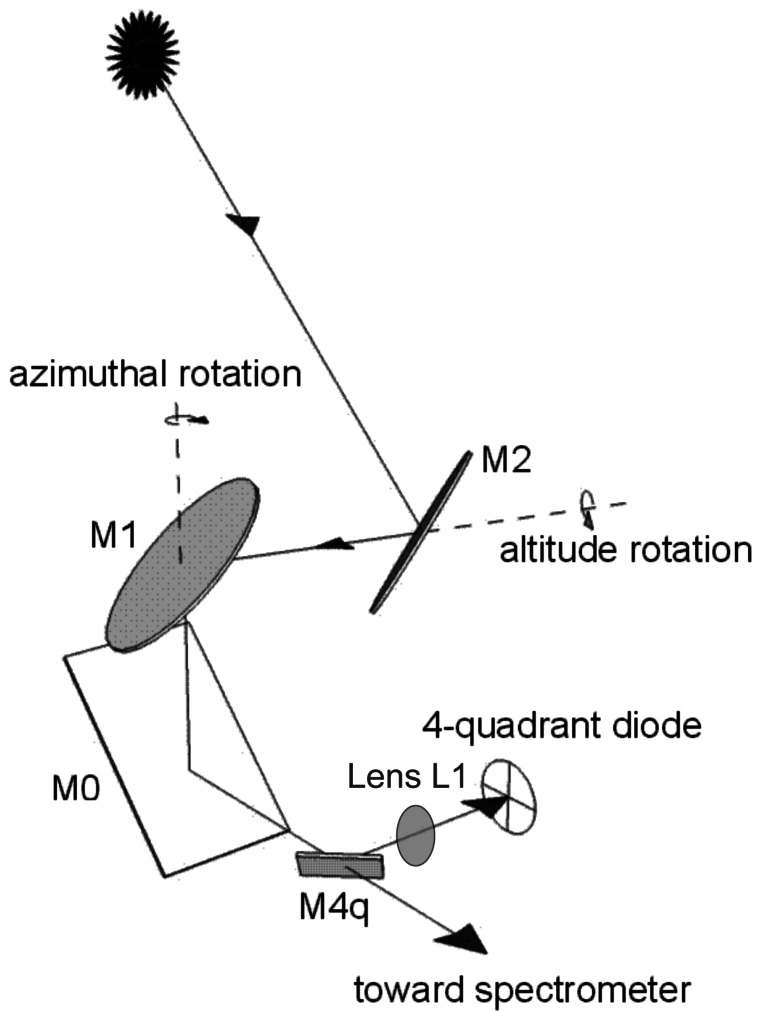
Geometrical setup of the considered solar tracker, using two 45-degree mirrors, M1 and M2, rotating along orthogonal axes. Mirror M0 directs the Sun light into a spectrometer. A fraction of the light beam is deflected toward a 4-quadrant photodiode enabling a closed-loop control of the mirrors position.

Figure 1 shows a popular altazimuthal tracker design. It consists of two elliptical mirrors held in 45 degrees relative to the vertical, facing each other (M1 and M2). Both M1 and M2 rotate along the azimuthal axis and M2 rotates as well around a horizontal axis (altitude direction). M0, and possibly other fixed mirrors, direct the light beam into the spectrometer optical axis. A 4-quadrant photodiode is used as a position sensor for a closed-loop control of the mirrors position once their positioning towards the Sun has been set with enough accuracy, *i.e.*, once the Sun's image is visible by the photodiode. This altazimuthal setup is used with FTIR systems, e.g., in Kiruna [10] and Park Falls, Wisconsin [11]; it has been installed in Harestua to replace the equatorially mounted system [12]. Compact versions have also been developed for field campaigns [13,14]. A commercial version is sold by Bruker to be installed on their FTIR spectrometers [15]. A recent progress in the pointing accuracy has been reported [16], replacing the traditional quadrant diode with a CCD camera, but the problems discussed hereafter remain the same.

Because developing a solar tracker is typically a master's thesis work [10,13,14], technical implementations are difficult to access in the literature. Some more information is available about the systems used in solar energy applications but their geometries differ [17–20]. Someone building a Sun tracker can quickly find ephemeris calculations in many programming languages, but other issues arise quickly. It is first necessary to characterize the field-of-view (FOV) of the 4-quadrant diode in the considered optical design. This serves two purposes: determining the accuracy needed for the ephemeris's algorithm and making sure this FOV is larger than the Sun's apparent diameter (9 mrad). This last point is important to track constantly the center of the Sun, which reduces the uncertainties in the air mass factor and avoids Doppler shifts on the edges of the Sun ([16]). A second problem lies in the correction of the tracker orientation compared to the altazimuthal system in which the ephemeris is given, necessary for the calculated mode if the base of the solar tracker is not leveled. Thirdly, the relationship between the quadrant signal and the correction to apply on the mirrors positions depends on the tracker position itself [16]. Understanding this relationship is compulsory to achieve a smooth tracking. This article deals with these three problems successively.

## 2. Theoretical Basis

### 2.1. Ephemeris Accuracy and Field of View of the 4-Quadrant Diode

Calculating the Sun position in the sky given the time of observation and the geographical coordinates is well documented. A reference algorithm is given by Jean Meeus in [21], for which C([22]) or Matlab (http://www.mathworks.com/matlabcentral/fileexchange/4605-sunposition-m) versions are available. This accuracy degrades with larger zenithal angle due to atmospheric refraction, which depends on local meteorological conditions (for an accurate and wavelength dependent refraction formula, see [23]). Irregular variations in Earth rotation also limit the accuracy of ephemerids independently of refraction. On the other hand, the absolute accuracy of commercial rotation stages used in Sun trackers, e.g., Newport RV-160, is only 0.01°. This reduces the interest of using an accurate but complex algorithm for ephemeris's calculation, which is anyway not necessary providing a closed-loop control is performed on the mirrors' position. In this case, the lowest acceptable accuracy is thus determined by the field of view of the 4-quadrant diode: once the Sun image hits the quadrant, the tracking can be performed in closed-loop.

**Figure 2.**
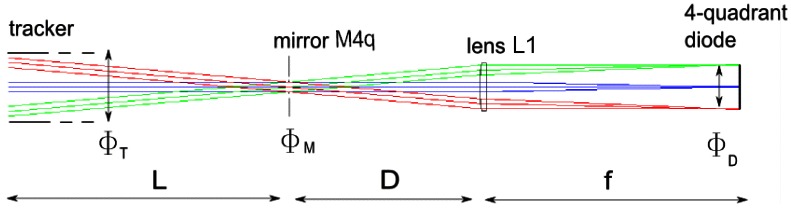
Optical scheme from the tracker mirrors to the 4-quadrant diode. The field of view seen by the diode depends on the different aperture sizes and path lengths. The green and red beams represent the Sun light path when the tracker is not perfectly aligned on the Sun.

Figure 2 shows the typical optical scheme between the tracker and the quadrant diode, the optical axis has been aligned for the sake of clarity. The distance L is measured from the first mirror which reflects the sunlight, *i.e.*, M2 in Figure 2. Close to the spectrometer, a part of the beam from the tracker, with diameter Φ*_T_* corresponding to the small axis of M1 and M2 on Figure 2, is deflected by a mirror of diameter Φ*_M_* to a lens which focuses the beam onto the quadrant. The maximum field-of-view seen by the diode (*FOV*_1_) depends on the focal length of the lens (*f*) and on the diameter of the quadrant (Φ*_D_*), according to ${\text{FOV}}_{1}=\arctan\frac{\Phi_{D}}{f}$. The lens L1 is unlikely to reduce the FOV assuming its size superior to the mirror's one. Indeed, the beam is parallel before the lens which implies that distance D can be reduced if necessary. The tracker aperture, in our case defined by the azimuthal stage free aperture, is more important, especially since the diameter of a rotation stage is limited and the distance between the tracker and the deflecting mirror (M4) depends on the observatory configuration. The mirror's FOV due to the tracker is ${\text{FOV}}_{2}=\arctan\frac{\Phi_{T}}{L}$.

Considering our set-up in Brussels, which is a typical FTIR station, the tracker's mirrors are 10 cm (Φ*_T_*) wide and the optical path to the spectrometer (*L*) 5 m long, which leads to a *FOV*_2_ of 20 mrad. On the other hand, the quadrant diameter (Φ*_D_*) is 6 mm while the focal length of the lens ((*f*)) is 200 mm, which gives 30 mrad for *FOV*_1_. The actual FOV is the minimum, 20 mrad, limited by the tracker size. This value is superior to the apparent diameter of the Solar disk which is important to track the center of the Sun. A simple algorithm can achieve such an accuracy for the ephemeris calculation, like the one given in the appendix. It is however necessary to take into account the orientation of the tracker, which can lead to pointing errors superior to the FOV.

### 2.2. Correcting the Tracker Orientation

One source of error, when pointing to the calculated Sun position, is the orientation of the baseplate of the tracker compared to the altazimuthal system. Depending on the observatory configuration, it may be difficult or impossible to align accurately the tracker along the North-South direction. If it was the only problem, the remaining constant offset could be simply fitted and added in the calculated azimuth. However, because the baseplate is never completely leveled either, other offsets are added to the calculated positions, affecting both azimuth and elevation in a way that depends on the pointing direction of the tracker. Some tracker uses an active search method to solve this problem. In practice they reach the calculated position and achieve spiral motion around this point to set the sun spot in the field-of-view of the sensor. Misalignment effect can on the other hand be taken into account in the calculation requires determining the Euler angles of the observatory and the tracker baseplate, respectively, compared to the azimuthal system. We discuss the Euler angles before describing our way to determine them. For the sake of simplicity, we only mention the observatory in the following, considering that the baseplate to be part of it.

**Figure 3.**
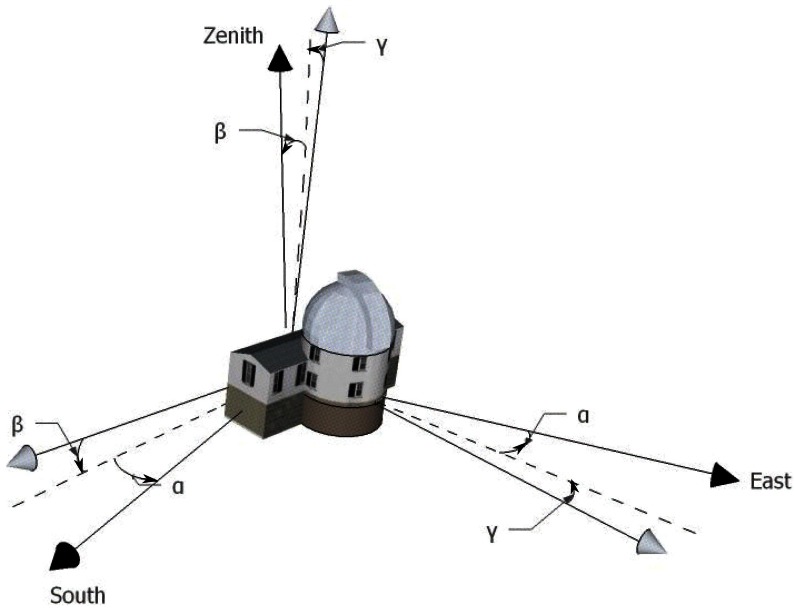
Illustration of the Euler Angles of an observatory compared to the altazimuthal coordinate system.

Euler angles of an observatory may be seen, as in Figure 3, as consecutive rotations around three orthogonal axes needed to account for the pitch, roll and yaw of this observatory Converting the solar altitude and azimuth to the observatory frame requires thus to compute the multiplication of three rotation matrices along the different axes, *i.e., R_x_*(*γ*), *R_y_*(*β*) and *R_z_*(*α*). The resulting matrix *M_offset_* expresses the transformation of coordinates due to the Euler angles.

(1) $$M_{\text{offsets}}=\left[\begin{array}{ccc} 1 & 0 & 0\\ 0 & \cos\gamma & -\sin\gamma \\ 0 & \sin\gamma & \cos\gamma \end{array}\right]\times \left[\begin{array}{ccc} \cos\beta & 0 & \sin\beta \\ 0 & 1 & 0\\ -\sin\beta & 0 & \cos\beta \end{array}\right]\times \left[\begin{array}{ccc} \cos\alpha & -\sin\alpha & 0\\ \sin\alpha & \cos\alpha & 0\\ 0 & 0 & 1 \end{array}\right]$$

The calculations leads to:

(2) $$M_{\text{offsets}}=\left[\begin{array}{ccc} \cos\alpha \cos\beta & -\sin\alpha \cos\beta & \sin\beta \\ \cos\alpha \sin\beta \sin\gamma +\sin\alpha \cos(\gamma ) & \cos\alpha \cos\gamma -\sin\alpha \sin\beta \sin\gamma & -\cos\beta \sin\gamma \\ \sin\alpha \sin\gamma -\cos\alpha \sin\beta \cos\gamma & \cos\alpha \sin\gamma +\sin\alpha \sin\beta \cos\gamma & \cos\beta \cos\gamma \end{array}\right]$$

In Cartesian coordinates, the unit vector (*x_t_,y_t_,z_t_*) giving the direction of the Sun in the observatory frame will thus be related to the solar spherical coordinates(*az*_0_,*alt*_0_) in the altazimuthal system:

(3) $$\left[\begin{array}{c} x_{t}\\ y_{t}\\ z_{t} \end{array}\right]=M_{\text{offsets}}\times \left[\begin{array}{c} \cos{\text{alt}}_{0}\cos{\text{az}}_{0}\\ \cos{\text{alt}}_{0}\sin{\text{az}}_{0}\\ \sin{\text{alt}}_{0} \end{array}\right]$$

Substituting *M_offset_* with Equation (2) we get the following expressions for those coordinates:

(4) $$\{\begin{array}{l} x_{t}=\cos(\alpha +{\text{az}}_{0})\cos\beta \cos{\text{alt}}_{0}+\sin\beta \sin{\text{alt}}_{0}\\ y_{t}=(\cos(\alpha +{\text{az}}_{0})\sin\beta \sin\gamma +\sin(\alpha +{\text{az}}_{0})\cos\gamma )\cos{\text{alt}}_{0}-\cos\beta \sin\gamma \sin{\text{alt}}_{0}\\ z_{t}=(\sin(\alpha +{\text{az}}_{0})\sin\gamma -\cos(\alpha +{\text{az}}_{0})\sin\beta \cos\gamma )\cos{\text{alt}}_{0}+\cos\beta \cos\gamma \sin{\text{alt}}_{0} \end{array}$$

These new Cartesian coordinates can then be converted to altitude (*alt_t_*) and azimuth (*αz_t_*) angles relative to the tracker:

(5) $$\{\begin{array}{ll} \rho_{t} & =\sqrt{x_{t}^{2}+y_{t}^{2}}\\ {\text{alt}}_{0} & ={\text{atan}}_{2}(z_{t},\rho_{t})\\ az_{t} & ={\text{atan}}_{2}(y_{t},x_{t}) \end{array}$$

In the above equation, *atan*_2_(*y, x*), available in many programming languages, stands for the argument of the complex number *x* + *iy*. It is closely related to the arctangent of *y/x* but it indicates unambiguously the quadrant of this angle on the trigonometric circle.

Determining Euler angles accurately by measurements is not easy. An analytical method to estimate them is given in [17] which basically consists of recording the position of the tracker at three different times and solving Equation (4). This is appropriate for the studied case, *i.e.*, a collector for solar energy application installed outside with only one mirror and no closed-loop control. With our considered two-mirror tracker, which does not collect light but directs it toward a spectrometer, other sources of misalignments appear. Indeed, the tracker is also likely to be misaligned compared to the spectrometer, and mirror themselves can be tilted. Other angles can be considered in *M_offsets_* and is done in [10]. In practical applications, despite the three Euler angles, the calculated mode is likely able to reach the Sun within the FOV of the 4-quadrant diode. With the closed-loop control it is easy to track the Sun during a whole clear-sky day providing an operator correctly sets the Sun tracker initially. Euler angles can then be fitted using all the recorded positions of the mirrors during the day. It has the advantage that other sources of misalignment are included: even if only three angles are fitted which may not exactly be the Euler angles, they minimize simultaneously the effects of all offsets. We implement this method in Section 4. This requires the closed-loop control of the tracker on the Sun position.

### 2.3. Ray Tracing in the Tracker

The photodiode signal indicates that the Sun beam is tilted compared to the optical axis of the spectrometer. The photodiode signals must be converted into angular movements of the altitude and azimuth axes of the tracker to correct the misalignment. If the photodiode was placed on the reference frame of the mirror M2 this conversion would be straightforward, but due to its position after the tracker it depends on the position of the tracker mirrors. A trial-and-error method to correct the misalignment is theoretically possible using analogue electronics without a computer but a smoother tracking can be achieved if the conversion is understood.

The conversion can be expressed once again by a matrix, which transforms in this case a vector hitting mirror M1 to a vector pointing to a direction in the sky given by its altitude and azimuth. It is the opposite of the light direction but is simpler to figure out, and considering Fermat principle, yields the same information.

The rotation of the two motorized stages can be accounted for using rotation matrices as described in the previous section. The reflection on the two mirrors is modeled using another matrix which takes the form:

(6) $$M=I-2nn^{T}$$

where I is the identity matrix and *n* the normal vector to the mirror surface. At reference position, the mirror are parallel and thus their normal is the same, given by the vector $\left(0,\frac{1}{\sqrt{2}},-\frac{1}{\sqrt{2}}\right)$. The transformation matrix for the two mirror is thus the same, *M_R_*, which is, from Equation (6):

$$M_{R}=\left[\begin{array}{ccc} 1 & 0 & 0\\ 0 & 0 & 1\\ 0 & 1 & 0 \end{array}\right]$$

**Figure 4.**
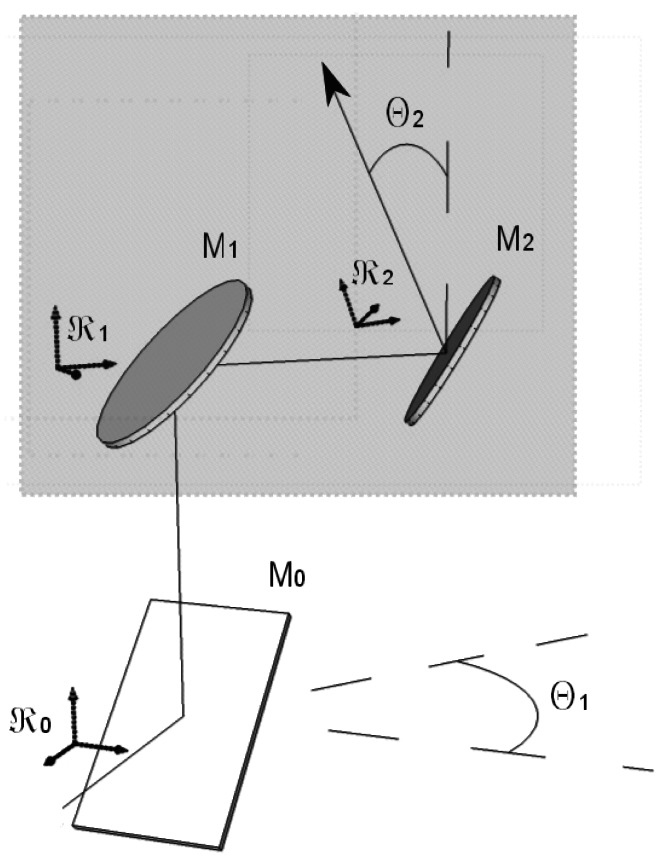
The tracker mirrors and their rotation can be modeled as rotation matrices in their reference frames, which apply to the beam vector. Note that the *z* and *y* axes are the same respectively for (ℜ_0_, ℜ_1_) and (ℜ_1_, ℜ_2_).

Figure 4 presents the tracker pointing to an azimuth *θ*_1_ and a zenith angle of *θ*_2_. The reference frames ℜ_1_ and ℜ_2_ are respectively attached to the mirrors *M*_1_ and M_2_, with the *x* axes in the direction of their small semi-axes and the *y* axes along the line joining the two mirrors. The optical system inside the frame, with only mirrors *M*_1_ and *M*_2_, can be expressed as a transformation whose matrix *M_tracker_* is:

(7) $$M_{\text{tracker}}=R_{z}(\theta_{1})\times R_{y}(\theta_{2})\times M_{R}\times R_{y}(-\theta_{2})\times M_{R}\times R_{z}(-\theta_{1})$$

The above formula is derived as follow: (a) the reflection on *M*_2_ (*M_R_*) is expressed in the reference frame of ℜ_1_ with a change of basis involving *R_y_*(−*θ*_2_); (b) this product of three matrices is multiplied on its right side by the preceding (seen from the spectrometer) reflection on *M*_1_(*M_R_*); (c) another change of basis is performed to express the transformation in ℜ_0_, involving *R_z_*(−*θ*_1_). *i.e.*,

(8) $$M_{\text{tracker}}=\left[\begin{array}{ccc} \cos\theta_{1} & -\sin\theta_{1} & 0\\ \sin\theta_{1} & \cos\theta_{1} & 0\\ 0 & 0 & 1 \end{array}\right]\times \left[\begin{array}{ccc} \cos\theta_{2} & 0 & \sin\theta_{2}\\ 0 & 1 & 0\\ -\sin\theta_{2} & 0 & \cos\theta_{2} \end{array}\right]\times \left[\begin{array}{ccc} 1 & 0 & 0\\ 0 & 0 & 1\\ 0 & 1 & 0 \end{array}\right]\times \left[\begin{array}{ccc} \cos\theta_{2} & 0 & -\sin\theta_{2}\\ 0 & 1 & 0\\ \sin\theta_{2} & 0 & \cos\theta_{2} \end{array}\right]\times \left[\begin{array}{ccc} 1 & 0 & 0\\ 0 & 0 & 1\\ 0 & 1 & 0 \end{array}\right]\times \left[\begin{array}{ccc} \cos\theta_{1} & \sin\theta_{1} & 0\\ -\sin\theta_{1} & \cos\theta_{1} & 0\\ 0 & 0 & 1 \end{array}\right]$$

Developing the matrix product yields the matrix of the tracker optical system as a function of the tracker position (*θ*_1_*,θ*_2_):

$$M_{\text{tracker}}=\left[\begin{array}{ccc} \cos\theta 1\cos\theta 2\cos(\theta_{1}-\theta_{2})+\sin\theta_{1}\sin(\theta_{1}-\theta_{2}) & \cos\theta_{1}\cos\theta_{2}\sin(\theta_{1}-\theta_{2})-\sin\theta_{1}\cos(\theta_{1}-\theta_{2}) & \cos\theta_{1}\sin\theta_{2}\\ \sin\theta_{1}\cos\theta_{2}\cos(\theta_{1}-\theta_{2})-\cos\theta_{1}\sin(\theta_{1}-\theta_{2}) & \cos\theta_{1}\cos(\theta_{1}-\theta_{2})+\sin\theta_{1}\cos\theta_{2}\sin(\theta_{1}-\theta_{2}) & \sin\theta_{1}\cos\theta_{2}\\ -\sin\theta_{2}\cos\theta_{1}-\theta_{2} & -\sin\theta_{2}\sin\theta_{1}-\theta_{2} & \cos\theta_{2} \end{array}\right]$$

**Figure 5.**
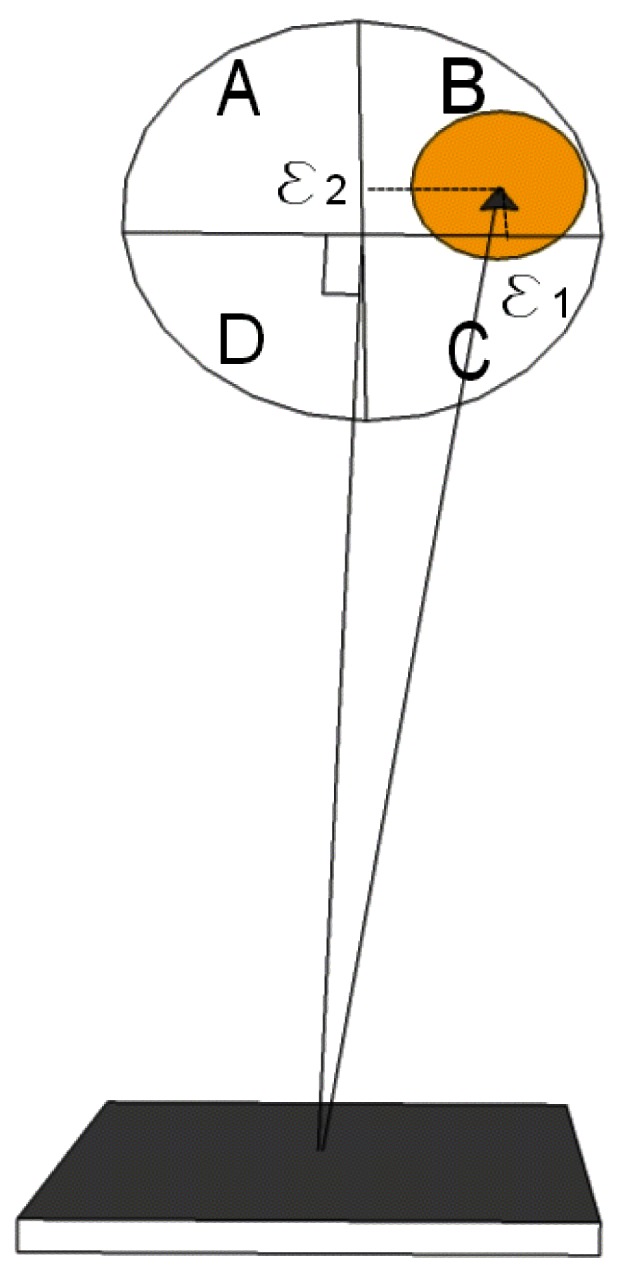
Sun spot hitting the quadrant, not to scale.

The transformation expressed by *M_tracker_* can now be applied to a vector corresponding to the Sun light beam direction on the spectrometer side of the tracker. It will lead to the position of the Sun in Cartesian coordinates. The vector is built from the 4 diode signals (VA,VB,VC,VD), as represented in Figure 5. Basically an offset position (*ε*_1_,*ε*_2_) is computed for the Sun spot on the diode plane compared to its center by:

(9) $$\{\begin{array}{c} {ɛ}_{1}=(V_{B}+V_{C})-(V_{A}+V_{D})\\ {ɛ}_{2}=(V_{A}+V_{B})-(V_{C}+V_{D}) \end{array}$$

The spot offset (*ε*_1_,*ε*_2_) defines 2 coordinates of the beam vector. The last one, Λ, should represent the distance from the diode to mirror M1. Multiplying *M_tracker_* by the quadrant vector (*ε*_1_,*ε*_2_,Λ) would yield accurate Sun angles after conversion to spherical coordinates, but is not practically possible with a diode, contrary to an imaging sensor. The calculated position (*x_s_,y_s_,z_s_*) hereafter is thus not absolute but is sufficient to get the sign of the rotations to apply on the axes. Λ can be chosen arbitrarily as long as its absolute value is large enough compared to *ε*_1_ and *ε*_2_. The solar pseudo-coordinates are then:

(10) $$\left[\begin{array}{c} x_{s}\\ y_{s}\\ z_{s} \end{array}\right]=M_{\text{tracker}}\times \left[\begin{array}{c} {ɛ}_{1}\\ {ɛ}_{2}\\ \Lambda \end{array}\right]$$

In practice, the quadrant vector may differ from (*ε*_1_,*ε*_2_,Λ) due to reflections such as on the mirrors M0 and M4q on Figure 1, necessary to deviate a part of the beam to the 4-quadrant photodiode. Defining the position vector thus requires to pay attention to the optical path from M1 to the photodiode. In section 4, we explain how we deal with the problem in our particular case.

Developing Equation (10) yields:

(11) $$\{\begin{array}{l} x_{s}=(\cos\theta_{1}\cos\theta_{2}\sin(\theta_{1}-\theta_{2})-\sin\theta_{1}\cos(\theta_{1}-\theta_{2})){ɛ}_{2}+(\sin\theta_{1}\sin(\theta_{1}-\theta_{2})+\cos\theta_{1}\cos\theta_{2}\cos(\theta_{1}-\theta_{2})){ɛ}_{1}+\Lambda \cos\theta_{1}\sin\theta_{2}\\ y_{s}=(\sin\theta_{1}\cos b\sin(\theta_{1}-\theta_{2})+\cos\theta_{1}\cos(\theta_{1}-\theta_{2})){ɛ}_{2}+(\sin\theta_{1}\cos\theta_{2}\cos(\theta_{1}-\theta_{2})-\cos\theta_{1}\sin(\theta_{1}-\theta_{2})){ɛ}_{1}+\Lambda \sin\theta_{1}\sin\theta_{2}\\ z_{s}=-\sin\theta_{2}\sin(\theta_{1}-\theta_{2}){ɛ}_{2}-\sin\theta_{2}\cos(\theta_{1}-\theta_{2}){ɛ}_{1}+\Lambda \cos\theta_{2} \end{array}$$

It is then possible to calculate roughly an altitude(*θ*_2_*_S_*) and azimuth(*θ*_1_*_S_*) for the Sun applying the Cartesian to spherical coordinates conversion (Equation (5)). This position is approximate and relative to the tracker since it does not take into account the Euler angles described in the last section, but what matters are the signs of the differences between these calculated values and the current altitude and azimuth relative to the tracker, defined by *θ*_1_ and *θ*_2_. The angular corrections to apply on the two axes are then:

(12) $$\{\begin{array}{c} d_{\theta 1}=\mathrm{sgn}(\theta_{1S}-\theta_{1})k_{1}\\ d_{\theta 2}=\mathrm{sgn}(\theta_{2S}-\theta_{2})k_{2} \end{array}$$

where *k*_1_ and *k_2_* are the tracking angle steps that should be small to have a smooth tracking, yet large enough for the mechanical resolution of the rotation stages and the apparent movement of the Sun. The azimuth changes for instance at a rate of 15° per hour, assuming 1 second between the steps, *k*_1_ should not be under 0.004°.

## 3. Automation Issues

From a control theory perspective, the altazimuthal tracker and its feedback is a non-linear multi-input multi-output (MIMO) system. Indeed, two outputs defining the pointing direction (*θ*_1_ and *θ_2_*) are controlled by two inputs, *i.e.*, the coordinates of the Sun spot on the photodiode(*ε*_1_ and *ε*_2_), and the relationship between the inputs and the outputs varies with the position of the tracker. However, having modeled this relationship in the previous section, it is possible to change the feedback scheme while tracking. In control theory, this is an example of adaptive control.

The correction of the azimuth and altitude angles discussed earlier only takes into account the current error, *i.e.*, the tilt of the solar beam compared to the optical axis of the spectrometer. This is very coarse and can lead to oscillations. A proper feedback loop includes the derivative and integral of the error relative to the time as well, respectively to reduce the overshoot and the residual part of the errors. This involves to tune the three parameters of a proportional-integral-derivative (PID) controller. Considering the two outputs, the setup needs two PID controllers.

**Figure 6.**
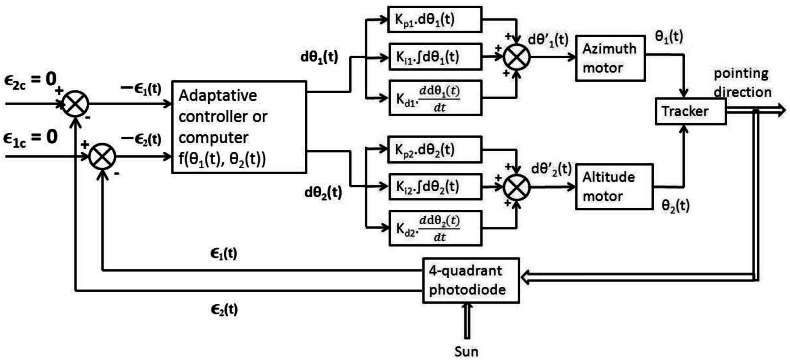
Control loop for an altazimuthal tracker.

Figure 6 suggests a feedback loop for the tracker. Note that *ε*_1_*_c_* and *ε*_2_*_c_* are null if the photodiode is correctly aligned compared to the spectrometer optical axis. The adaptive control algorithm on the figure (*f* (*θ*_1_(*t*), *θ*_2_(*t*))) originates from the formula derived in the previous section. There are 6 parameters to tune to optimize the feedback, which corresponds to the proportional, derivative and integral terms of the two PID controllers. Several methods exist to optimize PID parameters, with different complexity. We think the simple Ziegler-Nichols method could be appropriate. It consists in setting Ki and Kd to 0 and increasing Kp from 0 to the value Kpc with which oscillations occur at constant amplitude, with a period Tc. Three good values for Kp, Ki and Kd can then be derived as:

(13) $$\{\begin{array}{c} K_{p}=0.6K_{c}\\ K_{i}=\frac{2K_{p}}{T_{c}}\\ K_{d}=\frac{K_{p}T_{c}}{8} \end{array}$$

Considering the latitude of the Reunion Island observatory where the tracker is installed (20.9°S) it is worth considering an issue occurring with the altazimuthal geometry, *i.e.*, the singularity at zenith. As the altitude gets closer to zenith, the azimuth rotation gets more and more difficult to control. At Reunion Island, the maximum altitude is reached around November the 26th and January the 16th. Around this date, the measurement dead time can reach one hour. To our knowledge, there is no good solution to solve the problem. Nevertheless, to limit the measurement dead time around noon, we propose to implement in the adaptive controller another mode starting when the altitude is too high and the problem happens: shifting the azimuth mode from feedback controlled to calculated mode. The elevation is still controlled by the photodiode. This may not be accurate enough to keep the Sun spot in the spectrometer's iris, but it would at least avoid possible incoherent movement of the tracker.

## 4. Application for a FTIR Measurement Station

Our group has been doing FTIR measurements at Reunion Island for several years ([24–26]). The place is interesting since atmospheric measurements are sparse in the tropical and subtropical regions. Aiming at long-term monitoring and cost-effectiveness, a station at Saint-Denis (20.9°S, 55.5°E, 50 m a.s.l.) has been automated [9], which includes Sun tracking, meteorological logging and FTIR measurements with a Bruker 120M. The solar tracker currently used was developed at Denver University. Since September 2009, this station is officially part of the NDACC network and in Spring 2012 it will move to the new Maido Observatory (21.1°S, 55.4°E, 2200 m a.s.l.).

A second FTIR station has been installed in Saint-Denis in September 2012. This station, which is also automated, is based on a Bruker 125 HR spectrometer, more appropriate to measure CO_2_ atmospheric loading, in the framework of the new Total Carbon Column Observing Network (TCCON). The geometry of the Sun tracker is altazimuthal. It was built at our institute and used to validate the methods described in the last section.

**Figure 7.**
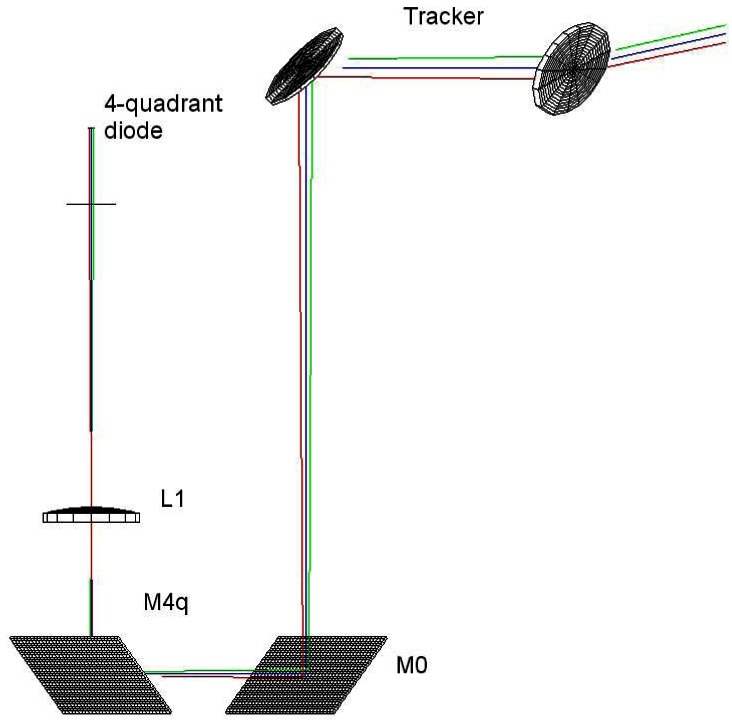
Optical paths of our particular set-up from the tracker to the 4-quadrant diode (not to scale).

This new solar tracker uses a Newport RV-160 rotation stage for the azimuth rotation and a Vexta stepping motor with a gear box for the altitude. Both rotations are driven by a Newport XPS controller, linked to the controlling PC. The tracker mirrors are elliptical with a 10 cm minor axis. The photodiode setup was purchased from Bruker with the spectrometer and is installed at the input window of the spectrometer. It consists of a 1 cm mirror which reflects a small portion of the incoming light to a 18 cm focal length lens which focuses the beam onto the 4-quadrant photodiode. The optical path is shown in Figure 7. The FOV of the 4-quadrant photodiode is 20 mrad (see Section 2.1). The algorithm used to compute the ephemeris is the one given in appendix. During operation, the mirrors positions are refreshed every half second according to the calculated position or to the signal on the 4-quadrant photodiode using the methods described in Section 2.3. From Figure 7, it is clear than the beam from the tracker undergoes two orthogonal reflections before hitting the photodiode, which we take into account multiplying *Mtracker* on its rigth side by the corresponding factors derived from Equation (6).

**Figure 8.**
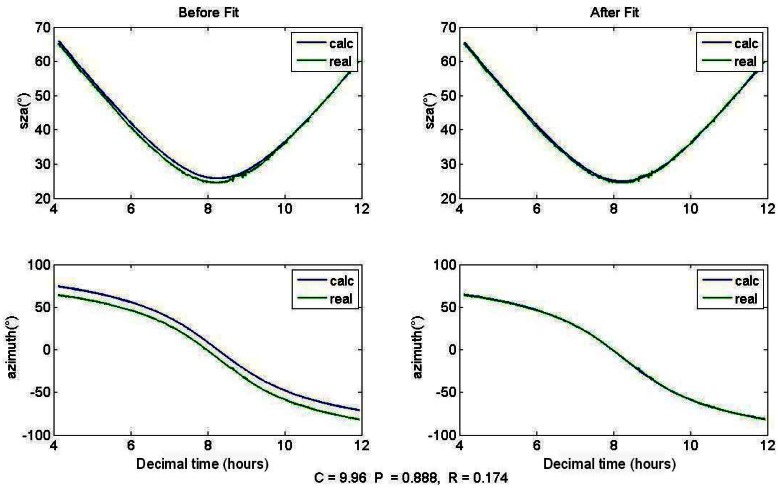
Fit of the Euler Angles to take into account the alignment offsets in the calculation mode. The track was performed on 12 September 2011.

Figure 8 shows a fit of the Euler Angles. The track was performed on 12 September 2011 in Saint-Denis. The left panel shows the calculated mirror positions neglecting Euler Angles, together with the actual ones when the active tracker was operational. Weather was clear-sky and enabled to record a long Sun path, demonstrating the capacity of the active tracking algorithm described in Section 2.3. Around one hundred points were extracted from the log file of the tracker position and used to fit the Euler Angles with an unconstrained nonlinear minimization. The three angles *α,β* and γ described in Section 2.2 were respectively estimated to be 9.96°, 0.888° and 0.174°. The right panel shows how these fitted angles improve the calculated Sun position. The maximum offset between calculated and actual position is now 0.5°, *i.e.*, 9 mrad, which is under the 20 mrad of the photodiode's FOV. Providing this accuracy in the calculated mode, the tracking system is able to set the Sun's image onto the 4-quadrant photodiode and then start the active tracking without the need of an operator.

## 5. Conclusions

We have derived the geometrical formulas needed to track the Sun with a kind of altazimuthal tracker widely used in atmospheric remote sensing. The setup is based on two rotating 45° mirrors facing each other and a 4-quadrant photodiode involved in a closed-loop control of the tracker. After discussing the required accuracy for the calculated mode and calculating the FOV of the sensor, we described how to take into account and estimate the Euler angles, representing the orientation of the tracker compared to the ground. These sections can actually be applied to other tracking setups. On the other hand, even if the method is general, the formula for the active tracking depends strongly on the optical configuration and may not be used for other trackers' geometries. We have proposed a control loop with PID to achieve a smooth tracking while reducing overshoot and the residual part of the error. Finally, we have tested the formulas with a custom-built solar tracker that has been installed together with a FTIR spectrometer at Reunion Island in September 2011.

Among the future work will be the improvement of the tracking smoothness, and particularly the tuning of the six parameters of the PID controllers. We will also implement the solution presented in Section 3 and check whether the measurement dead time can be reduced.

A characteristic of the Maido observatory is the very regular cloud cycle. At noon, the clouds reach the observatory almost every day. This is very convenient for clouds studies but less for solar occultation trace gases measurements. On the other hand, the nights are so clear up there that the observatory was first supposed to be dedicated to astronomical research. This is thus a good place to try Moon tracking and we plan to work on that in the future.

## Figures and Tables

**Figure A1.**
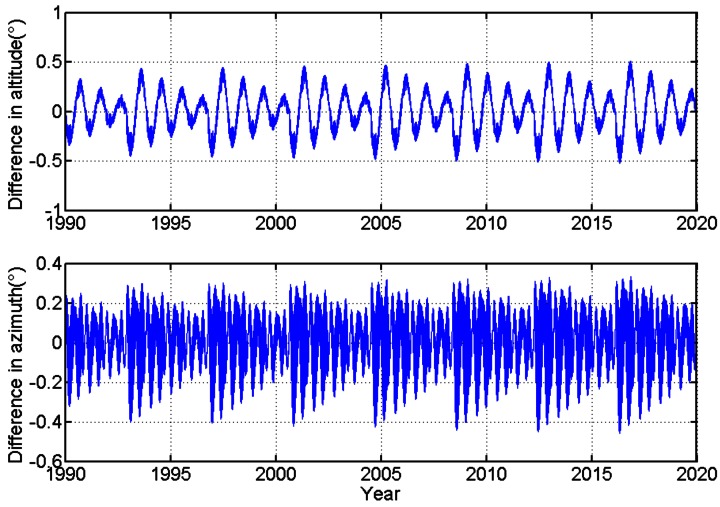
Comparison between the formulas reproduced in appendix and JPL ephemerides.

## A. Equations for Solar Ephemeris

For the sake of completeness, we reproduce here the simple algorithm we use to calculate the solar coordinates given a date and a position, taken from [27]. It should be accurate enough for most tracking purposes.

We first compute the fractional year (γ) in radians:

(14) $$\gamma =\frac{2\pi }{365}*(JD-1+\frac{T-12}{24})$$

where *JD* stands for the Julian day and *T* for the UTC decimal time.

Then we derive the equation of time (Δ*t*) in minutes:

(15) Δt=229.18∗(0.000075+0.001868cosγ−0.032077sinγ−0.014615cos2γ−0.040849sin2γ)

The equation of time represents the difference between apparent solar time and mean solar time, which can be as large as 16 min. It is due to the obliquity of the ecliptic and the elliptical form of the earth orbit.

From the fractional year, we also get the solar declination (*δ*_⊙_) in radians:

(16) $$\delta_{⊙}=0.006918-0.399912\cos\gamma +0.070257\sin\gamma -0.006758\cos2\gamma +0.000907\sin2\gamma -0.002697\cos3\gamma +0.00148\sin3\gamma$$

The declination is the equivalent of the latitude on the celestial sphere.

The offset *T_off_* (in minutes) between the UTC time and the true solar time depends on the longitude (in degrees East) and is:

(17) $$T_{\text{off}}=\Delta t-4*\text{longitude}$$

The true solar time (*tst*, in minutes) is then:

(18) $$\text{tst}=\text{hour}*60+\text{min}+\text{sec}/60+T_{\text{off}}$$

where *hour, min* and *sec* are the components of the UTC time.

The solar hour angle, in degrees, comes from the true solar time as:

(19) $$\text{ha}=\frac{\text{tst}}{4}-180$$

For a given latitude, the hour angle and the declination are converted to horizontal coordinates, i.e., solar zenith angle (*ϕ*) and azimuth (*θ*, from south positive eastwards) as:

(20) $$\phi =\arccos(\sin\text{lat}\sin\delta_{⊙}+\cos\text{lat}\cos\delta_{⊙}\cos\text{ha})$$

(21) $$\theta =-{\text{atan}}_{2}(\sin\text{ha}\cos\delta_{⊙},\cos\text{ha}\sin\text{lat}\cos\delta_{⊙}-\cos\text{lat}\sin\delta_{⊙})$$

Finally, the effect of refraction in arcminutes can be approximated using Sæmundsson's formula [21]:

(22) $$R=\frac{1.02}{\tan\;h+\frac{10.3}{h+5.1}}$$

where *h* is the unrefracted altitude in degree.

Figure A1 shows a comparison between the algorithm presented below and the Jet Propulsion Laboratory HORIZONS (http://ssd.jpl.nasa.gov/?horizons) ephemeris calculator. We compare the altitude and azimuth of the Sun seen from Brussels (50.85°N,4.35°E) over a 30-year period, between 1990 and 2020. Differences in altitude and azimuth are within 0.5°.
